# Microwave Heating for Synthesis of Carbonaceous Adsorbents for Removal of Toxic Organic and Inorganic Contaminants

**DOI:** 10.3390/molecules28196825

**Published:** 2023-09-27

**Authors:** Aleksandra Bazan-Wozniak, Katarzyna Machelak, Agnieszka Nosal-Wiercińska, Robert Pietrzak

**Affiliations:** 1 Department of Applied Chemistry, Faculty of Chemistry, Adam Mickiewicz University in Poznań, Uniwersytetu Poznańskiego 8, 61-614 Poznań, Poland; aleksandra.bazan@amu.edu.pl (A.B.-W.); katarzyna.machelak3@gmail.com (K.M.); 2 Department of Analytical Chemistry, Institute of Chemical Sciences, Faculty of Chemistry, Maria Curie-Sklodowska University in Lublin, Maria Curie-Sklodowska Sq. 3, 20-031 Lublin, Poland; agnieszka.nosal-wiercinska@mail.umcs.pl

**Keywords:** carbonaceous adsorbents, pyrolysis, microwave heating, NO_2_, methylene blue/malachite green, kinetic and thermodynamic study, water purification

## Abstract

The residues obtained from the extraction of *Inonotus obliquus* fungus were used to produce carbonaceous adsorbents. The initial material was subjected to pyrolysis in a microwave oven. The adsorbents were characterized through elemental analysis, low-temperature nitrogen adsorption/desorption isotherms, and Boehm titration. The carbonaceous adsorbents were tested for the removal of NO_2_, methylene blue, and malachite green. The results indicated that the obtained carbonaceous adsorbents exhibited basic characteristics and possessed specific surface areas of 372 and 502 m^2^/g. The adsorption process of liquid contaminants was modeled using the single-layer Langmuir model. The maximum adsorption capacities were found to be 101 and 109 mg/g for methylene blue, and 75 and 77 mg/g for malachite green. The kinetic study demonstrated that the adsorption of methylene blue and malachite green was better described by a pseudo-second order model. The study affirmed that the adsorption of organic dyes onto the resultant carbonaceous adsorbents was both spontaneous and endothermic. The study also demonstrated that the presence of an air stream during the NO_2_ adsorption process and prehumidization of the adsorbent with humid air had a beneficial effect on the obtained sorption capacities. In conclusion, the study demonstrated that pyrolysis of the extraction residues from the fungus *Inonotus obliquus* yields highly effective, environmentally friendly, and cost-efficient carbonaceous adsorbents for the removal of both gaseous and liquid pollutants.

## 1. Introduction

Over the last few decades, due to the ongoing expansion of industry and the widespread use of an increasing quantity and variety of chemicals, the environment has suffered significant damage [1]. This encompasses the introduction of synthetic fertilizers, pesticides, dyes, and various surfactants into the soil and surface waters. Moreover, our atmosphere has been polluted by nitrogen and sulfur compounds, carbon dioxide, and particulate pollutants [2,3]. It is becoming increasingly important to identify new and improved technologies for cleaning up the environment. As such, carbonaceous materials are now being utilized as effective adsorbents of liquid and gas-phase pollutants [4].

Carbonaceous materials can be derived from both agricultural waste and biomass, offering significant environmental benefits. The utilization of waste materials not only reduces pollution but also cuts down production costs for carbon adsorbents [5]. The selection of a precursor material for carbon adsorbent production is of utmost importance, as its characteristics dictate the physicochemical properties of the resulting materials and influence the choice of production process parameters [6]. The cost and suitability of a carbon adsorbent rely on the precursor utilized during its production. Hence, to acquire an adsorbent with specific features and intended purpose, it is imperative to select an appropriate initial raw material [7]. The physicochemical and sorption properties of carbon adsorbents are influenced by the method of their production, and currently, microwave heating has gained popularity as a viable option [8]. This method offers several advantages, including speed, selectivity, homogeneity, and precise process control. Furthermore, it enables the heating of the sample internally, leading to faster attainment of the desired processing temperature and reduced production costs due to lower time and energy inputs. Microwave radiation also promotes higher reaction efficiencies with fewer emissions of hazardous substances, making it an environmentally friendly choice [9,10].

Therefore, carbonaceous adsorbents were produced using residues obtained from the extraction of a fungus known as *Inonotus obliquus*. This fungus belongs to the *Hymenochaetaceae* family and typically thrives on the living trunks of mature birch trees. The subcortical spinneret of this fungus was isolated and found to contain polysaccharides with diverse pharmacological properties, including anticancer, antioxidant, anti-inflammatory, and immune-enhancing effects [11]. In this study, microwave heating was employed for the production of carbonaceous adsorbents. The resulting materials were then utilized for the removal of liquid pollutants such as methylene blue and malachite green, as well as gaseous pollutants—NO_2_.

## 2. Results and Discussion

### 2.1. Characterization of the Precursor and Adsorbents

Table 1 displayed the elemental composition and acid–base properties of both the starting material and the resulting carbonaceous adsorbents used in the study. According to the information presented in Table 1, it can be concluded that the raw material used had a low percentage of elemental carbon (55.5 wt.%) in its structure and a significant oxygen content exceeding 30 wt.%. The other elements present were in the following amounts: H^daf^—8.2 wt.%, N^daf^—2.9 wt.%, and S^daf^—0.1 wt.%. The study showed that the extraction residue of the fungus *Inonotus obliquus* contained 6.2% mineral matter by weight within its structure. Further analysis of the data provided in Table 1 indicated that the starting materials used exhibited a notable abundance of surface oxygen groups, primarily of an acidic nature rather than basic. This observation is corroborated by the slightly acidic reaction of the precursor’s aqueous solution.

The chemical composition and acid–base properties of the obtained carbonaceous adsorbents were also analyzed (Table 1). From the data in Table 1, it can be seen that the pyrolysis process of the raw material led to a significant increase in the carbon content in the structure of the obtained samples. It is worth noting that the adsorbent pyrolyzed at 600 °C contained the highest amount of elemental carbon. On the other hand, the oxygen content in the synthesized samples M5 and M6 ranged from 14.3 wt.% to 19.6 wt.%. Irrespective of the pyrolysis temperature, this process contributes to reduction in the hydrogen content and an increase in the amount of nitrogen in the structure of the carbon adsorbents compared to the starting raw material. In addition, the carbon materials tested contain more mineral matter than the starting material. Sample M5 contained 9.1 wt.% ash in its structure, while sample M6 contained 9.8 wt.% ash. Based on the Boehm titration results, it was discovered that the adsorbents exhibited a basic surface character, regardless of the pyrolysis temperature employed. Sample M6 exhibited the most enriched surface chemistry, with 3.3 mmol/g of oxygen groups, compared to 0.2 mmol/g of acid groups and 3.1 mmol/g of basic groups. In contrast, the M5 adsorbent was produced by pyrolysis of the original material at 500 °C, resulting in a configuration with 0.3 mmol/g acid groups and 2.8 mmol/g basic groups. The data presented in Table 2 indicate that higher pyrolysis temperatures facilitate the formation of basic functional groups [12]. The pH measurement of the water extracts for M5 and M6 samples corroborates the results of Boehm titration.

**Figure 1:**
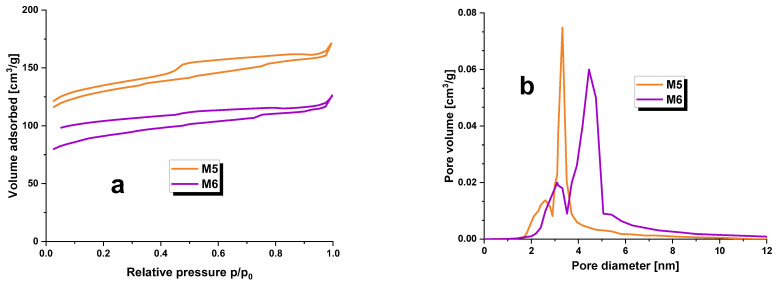
Low-temperature nitrogen adsorption/desorption isotherms (**a**) and pore size distribution (**b**) for carbonaceous adsorbents prepared.

Specific surface area measurements were carried out on the carbonaceous adsorbents obtained, and the outcomes are presented in Table 2 and visualized in Figure 1. The investigation included determining the specific surface area, micropore area, total pore volume, micropore volume, and average pore diameter. The S_BET_ values for samples M5 and M6 ranged between 372 and 502 m^2^/g. The carbonaceous adsorbent with the highest degree of surface development was M6, which also possessed the largest pore volume of 0.6 cm^3^/g. In addition, M6 exhibited the highest degree of microporosity compared to all other adsorbents of its kind. Furthermore, Table 2’s data analysis suggested that elevating pyrolysis temperatures by 100 °C results in favorable changes to the textural parameters of carbonaceous adsorbents. In light of the average pore diameter values for M5 and M6 adsorbents, which fall within the range of 3.2–3.7 nm, it can be deduced that the second type of pores found in the structure of the produced carbon materials was mesopores [13].

Additionally, Figure 1a exhibits low-temperature nitrogen adsorption/desorption isotherms for the obtained adsorbents. The shape of these isotherms is in line with type IV(a) isotherms according to the IUPAC classification. A hysteresis loop is visible in the curves, and a wide hysteresis loop suggests a major presence of mesopores in the structure of the carbonaceous adsorbents obtained during this study. In Figure 1a, the hysteresis does not close in the typical range of 0.3–0.4 p/p_0_; the mineral substance may act as a ballast to fill the porous structure of the carbonaceous adsorbents and cause poorer low temperature nitrogen adsorption/desorption results. The observed hysteresis loops belong to category H4 according to the IUPAC classification [14]. It should be noted that although samples M5 and M6 have a lower specific surface area, they still exhibit a more developed surface compared to activated carbons obtained from the physical activation of plant raw material residues [12] or the chemical activation of *Acacia erioloba* seed pods with H_2_SO_4_ [15].

### 2.2. Adsorption Study

Table 3 displays the sorption capacity of the obtained carbonaceous adsorbents towards NO_2_. Based on the data, it can be concluded that the efficiency of removing this toxic gas depended largely on the conditions of the adsorption process and the pyrolysis temperature. Among the adsorbents, M6 proved to be the most effective, with a sorption capacity ranging from 10.0 to 28.2 mg/g, depending on the adsorption variant. Conversely, the sorption capacity of the M5 sample ranged from 8.9 to 25.7 mg/g. Further analysis of the data in Table 3 reveals that prehumidization of the carbon bed for a duration of 30 min, prior to adsorption conducted in both dry and wet conditions, had a favorable impact on the quantity of nitric(IV) oxide adsorbed. In wet adsorption conditions, humidification of the bed enhanced the sorption capacity of the samples. The differences between the capacities obtained during adsorption before and after bed humidification were more notable than those observed in dry conditions. Data from Table 3 show that the NO_2_ removal efficiency of the adsorbents increased with higher pyrolysis temperatures.

**Figure 2:**
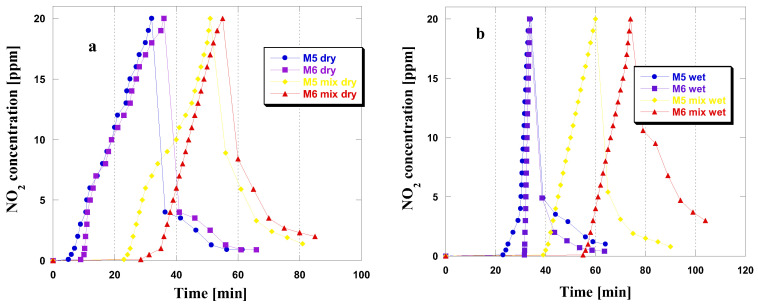
NO_2_ breakthrough curves for the carbonaceous adsorbents studied in dry/mix dry (**a**) and wet/mix wet (**b**) conditions.

Upon analyzing the curves presented in Figure 2, which depict changes in NO_2_ concentration during adsorption in four different variants, only minor differences were observed among the tested materials. This suggests a similarity in the adsorption mechanisms of these materials. Notably, from the shape of the curves, it is evident that the period of time where NO_2_ concentration is equal to or near zero is longer for both adsorbents in wet and mix wet conditions. Furthermore, an increase in NO_2_ concentration to 20 ppm was observed after reaching the breakthrough point of the bed. Prehumidization before the NO_2_ adsorption process in dry conditions extended the duration of time in which the NO_2_ concentration remained at or near zero. This observation is consistent with the results obtained from the adsorption process conducted under wet conditions, suggesting that water vapor has a beneficial effect on the efficiency of NO_2_ adsorption. The results indicate that water vapor positively affects NO_2_ adsorption efficiency, resulting in greater sorption capacities in such situations [12]. After reaching the NO_2_ concentration of 20 ppm and discontinuing its flow to the adsorbent bed, the NO_2_ concentration reduces. This may suggest that the majority of the adsorbed gas has been firmly attached in the porous structure or has undergone chemisorption [12]. This phenomenon is likely due to the significant quantity of basic functional groups on the surface of the obtained samples (Table 1). Only for the M6 sample under mix wet conditions did the concentration of NO_2_ remain at a relatively high level, even after 30 min of flushing the adsorbent bed with a stream of clean air.

The sorption capacities of methylene blue and malachite green for the carbonaceous adsorbents obtained in the experiment are shown in Table 4. The M6 adsorbent demonstrated the highest efficacy against the liquid pollutants tested, with an experimental sorption capacity of 107 mg/g for methylene blue and 76 mg/g for malachite green. In turn, in the case of the M5 sample, lower sorption capacities were observed at 100 mg/g for methylene blue and 73 mg/g for malachite green, respectively. These sorption capacities were found to be clearly correlated with the present carbonaceous adsorbents’ textural parameters (Table 2). Upon analyzing the q_e_ values, it can be concluded that on testing the sorbents, an increase in pyrolysis temperature results in a slight increase in sorption capacity towards the tested dye.

The adsorption process of methylene blue and malachite green onto the acquired carbonaceous adsorbents has been presented using four isotherm models. Table 4 lists the computed parameters and correlation coefficients of these models. Considering the computed R^2^ values for all four models, it can be inferred that the Langmuir isotherm model was the most suitable one for elucidating the experimental data. The R^2^ correlation coefficient values for this model varied from 0.997 to 0.999 (independent of the dye type tested), indicating uniform adsorption happening throughout the adsorbent’s entire surface. Furthermore, the maximum adsorption capacities for the Langmuir model (q_max_) closely matched the experimental values, indicating that this isotherm provides the most accurate description of methylene blue/malachite green adsorption on M5 and M6 samples. In discussing the available sites’ affinity and the free energy of adsorption, the Langmuir constant K_L_ is employed. For the carbonaceous adsorbents tested, the K_L_ ranged between 0.05 and 0.17 L/mg, indicating that the adsorption between the synthesized carbonaceous materials and the dye was a favorable process [16]. Considering the parameters calculated for the Freundlich model, it is noteworthy that higher correlation coefficient values were obtained during the adsorption of methylene blue. Specifically, the M5 adsorbent yielded a value of 0.961, and the M6 sample had a value of 0.979. This suggests that for these carbonaceous adsorbents, the adsorption process may also be multi-layered. Furthermore, the closer the value of the heterogeneity factor 1/n is to zero, the stronger the attraction between the adsorbate and the adsorbent surface. For the investigated samples (independent of the absorbed dye), this value ranged from 0.04 to 0.27, indicating significant surface heterogeneity. The Temkin isotherm theory posits that with increased surface coverage of the carbon adsorbent, the adsorption heat decreases. This correlation arises from the even dispersion of binding energy on the adsorbent surface and the interactions between the adsorbate and the adsorbent throughout the adsorption process. The higher value of parameter B for samples M5 and M6 during malachite green adsorption suggests a more advantageous interaction between the adsorbent and the dye, in contrast to their interaction with methylene blue. The study utilized the Dubinin–Radushkevich model to ascertain whether the adsorption process was predominantly physical or chemical. The calculated R^2^ coefficient values for this model ranged from 0.922 to 0.993, indicating a good fit. The E-value, a crucial parameter of this isotherm, signifies the energy associated with the chemical exchange of ions between the adsorbent surface and the dye. If it falls within the 8–16 kJ/mol range, it suggests a chemical interaction. If the energy exceeds 16 kJ/mol, the adsorption is considered chemical in nature, while values below 8 kJ/mol indicate adsorption driven by physical forces such as hydrogen bonds and van der Waals forces [17]. The outcomes of the E parameter, as presented in Table 4, indicate a primarily physical nature of adsorption.

**Figure 3:**
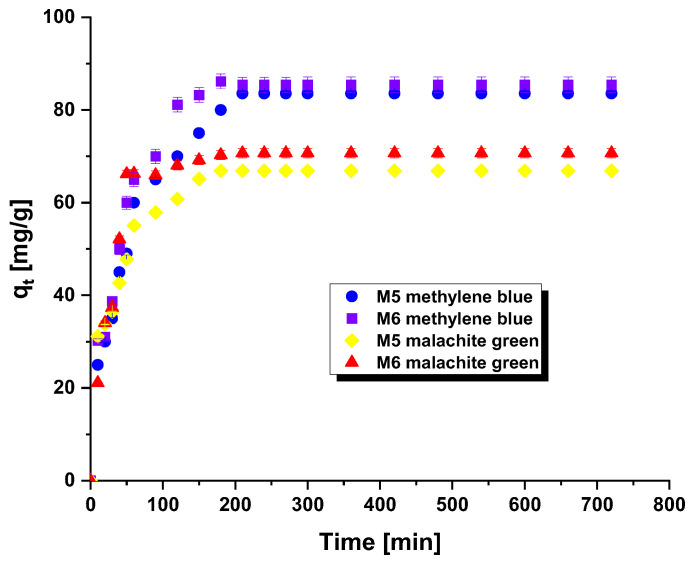
Effect of contact time on the adsorption of methylene blue/malachite green on the carbonaceous adsorbents.

Figure 3 depicted the influence of contact time between methylene blue/malachite green and carbonaceous adsorbents on the attained sorption capabilities. This element constitutes a crucial aspect in the wastewater treatment process. An analogous correlation was identified in both scenarios, where the sorption capability were augmented with prolonged adsorption time. This trend indicates that the active sites of the adsorbent are readily accessible, resulting in swift binding of the adsorbate. This can be particularly beneficial for wastewater treatment as it reduces the duration of the processing time [7].

Two kinetic models were employed to analyze experimental data related to the adsorption of methylene blue and malachite green on the surface of carbonaceous adsorbents (Table 5). The experimental data indicated that the pseudo-second order kinetic model most accurately describes the observations, with R^2^ values ranging from 0.995 to 0.999. This model is typically employed to elucidate adsorption processes controlled by chemisorption. In chemisorption, the functional groups in a dye either exchange or share electrons with the functional groups present on the surface of the adsorbents [16]. Furthermore, there were no significant differences between the theoretical and experimental amounts of methylene blue and malachite green adsorbed onto the tested adsorbents at equilibrium.

**Figure 4:**
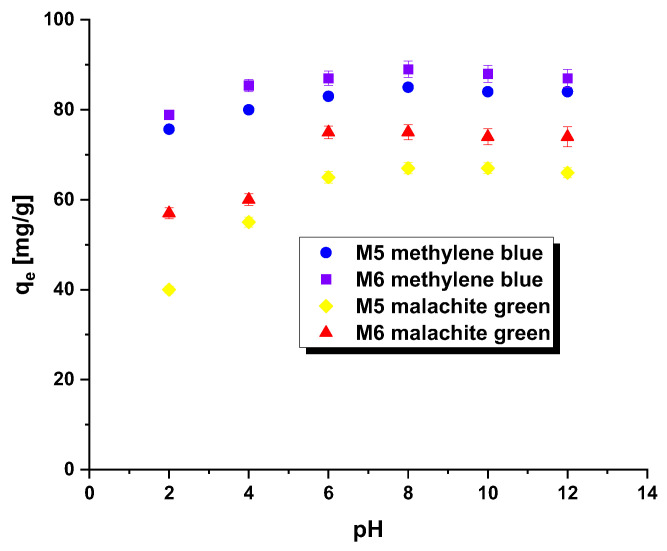
Effect of pH on the adsorption of methylene blue/malachite green on the carbonaceous adsorbents.

The pH_pzc_ parameter is instrumental in determining the optimal pH for adsorption experiments. When the system’s pH surpasses the pH_pzc_ value, the adsorbent surface acquires a negative charge, enabling the attraction of positively charged methylene blue/malachite green. Conversely, when the pH falls below the pH_pzc_, the adsorbent surface becomes positively charged, facilitating effective interaction with negatively charged dye [18]. In this study, the tested adsorbents demonstrated a pH_pzc_ of 7.1 for M5 and 7.3 for M6. This indicates that the system’s pH should be maintained above these pH_pzc_ values to ensure a negatively charged surface of the carbonaceous adsorbents, thus enhancing the adsorption of the tested pollutants. Additionally, it is feasible to form a covalent bond between hydroxyl ions and the dye cation. The impact of dye solution pH on the efficacy of carbonaceous adsorbent removal was assessed within the pH range of 2 to 12, as evident in Figure 4. Across both samples (regardless of the dye used), a noticeable increase in the q_e_ value was recorded for solutions within the pH range of 2 to 8, followed by a stabilization of sorption capacity levels. Notably, the removal of malachite green by means of samples M5 and M6 proved particularly sensitive to changes in dye solution pH.

Thermodynamic studies provide insight into whether the interaction between carbonaceous adsorbents and dye takes place via physisorption or chemisorption. Results presented in Table 6 show that as the process temperature increased, the sorption capacity towards the aqueous solution of methylene blue/malachite green rose. However, an even greater impact of this parameter was observed during the adsorption of malachite green on samples M5 and M6. The negative values of the ∆G° parameter indicate that the process was spontaneous. On the other hand, the positive values of enthalpy and entropy indicate that the adsorption processes studied are endothermic. The results of the ΔH° parameter range from 7.4 to 25.4 kJ/mol, indicating a physisorption process. The ΔS° parameter ranges from 45.6 to 96.0 kJ/mol. The higher the value of ∆S°, the more efficiently the process takes place [5]. Therefore, considering the results presented in Table 6, it can be concluded that the most efficient adsorption process occurs for malachite green and sample M5.

Table 7 illustrates the comparison between our study’s findings and other reports on the adsorption of NO_2_, methylene blue, and malachite green. The data suggest that the carbonaceous adsorbents extracted from *Inonotus obliquus* mushroom residues can effectively eliminate gaseous and liquid pollutants. Moreover, post thermochemical processing, the sorption capacity of these adsorbents is significantly higher than that used in this study. The sorption capacity of the M6 sample was found to be over two times less than that of biochar produced through the direct activation of nettle seed in a traditional furnace [5]. Ghouma et al. [19] showed a notably higher sorption capacity for NO_2_. In their research, the authors acquired activated carbon from olive stones via conventional two-stage physical activation. The carbon, obtained from waste tires via potassium hydroxide activation, had the ability to adsorb 11.44 mg/g NO_2_ in dry conditions [20]. Based on the NO_2_ results displayed in Table 7, it can be presupposed that the physical or chemical activation of the carbonaceous adsorbents produced in this study would lead to adsorbents with a considerably higher NO_2_ sorption capacity. It is imperative to grasp the reaction mechanism between the adsorbent and the adsorbate. In accordance with the information provided in references [12], it is anticipated that the adsorption of NO_2_ under dry conditions follows the subsequent mechanism:

–C* + NO_2_ → C(O) + NO

–C(O) + NO_2_ → –C(ONO_2_)

–C–C(ONO_2_) → CO_2_ + NO + –C*

–C–C(ONO_2_) → CO + NO_2_ + –C*

where: C* represents a carbon active site and –C(O) carbon oxygen complexes. Broadly speaking, the oxidation process of the carbon surface takes place as outlined by the reactions:

C + 2NO_2_ → CO_2_ + 2NO

C + NO_2_ → CO + NO

During adsorption under wet conditions, the reaction between nitrogen dioxide and water can result in the formation of nitric acid or a combination of nitric and nitrous acids:

3NO_2_ + H_2_O → 2HNO_3_ + NO

2NO_2_ + H_2_O → HNO_3_ + HNO_2_

which augments the sorption capacity for NO_2_ in the examined carbonaceous adsorbent.

The highest sorption capacity of the M6 sample for methylene blue was compared to results obtained from adsorbents derived from low-cost materials [21,22,23]. Carbon adsorbents were created through the chemical activation of orange and lemon peels [21], Maghara coal [22], and *Sargassum* (sp.) [23]. It is worth noting that the chemical activation process is considerably more expensive than the pyrolysis applied in this study since it necessitates the employment of activating agents such as potassium and sodium hydroxides, as well as inorganic acids such as phosphoric acid (H_3_PO_4_) or sulfuric acid (H_2_SO_4_). Analysis of the data summarized in Table 7 enables us to infer that adsorbents derived from orange and lemon peels and Maghara coal exhibited significantly lower methylene blue removal efficiency in comparison to carbonaceous adsorbent M6 [21,22]. Conversely, an adsorbent acquired from *Sargassum* (sp.) demonstrated more than double the sorption capacity towards the studied dye (223 mg/g) [23].

Analysis of the adsorbents’ ability to absorb malachite green reveals that biochar made from plantain leaves activated with H_3_PO_4_ [24] showed superior effectiveness in removing the dye, as presented in Table 7, compared to sample M6. In contrast, the carbonaceous adsorbent produced from the pyrolysis of extraction residue from *Inonotus obliquus* exhibited a sorption capacity comparable to that of activated carbon created by thermally processing date stones with nitric acid, exhibiting a sorption capacity of 98 mg/g [25]. On the other hand, the *Limonia acidissima* adsorbent (34.56 mg/g) exhibited almost half of the sorption ability towards malachite green when compared to the M6 sample [26].

The adsorption capacity of a carbonaceous adsorbent is determined by the interactions between the adsorbate and the adsorbent. The structures of methylene blue and malachite green indicate that hydrophobic interactions, hydrogen interactions, π–π interactions, and acid–base interactions are all possible. Based on the dye structure, it can be inferred that there are π–π interactions between the carbonaceous adsorbent and the aromatic structure of methylene blue/malachite green [22,24]. Additionally, M5 and M6 samples have a negatively charged surface, which enhances the electrostatic attraction between the positive adsorbate molecules and adsorbent molecules, ultimately leading to increased dye adsorption. The dyes utilized in this study were cationic, and thus, exist as positively charged ions in aqueous solution. Adsorption of the cationic dye is preferable at pH levels that are higher than pH_pzc_, owing to the existence of basic functional groups on the M5/M6 samples. Dye solutions with pH greater than 7.1 for sample M5 and 7.3 for adsorbent M6 are favorable circumstances for the adsorption of the investigated pollutants, as previously explained [22,24].

## 3. Materials and Methods

### 3.1. Materials

Methylene blue and malachite green as model adsorbates were purchased from Avantor Performance Materials Poland S.A. (Gliwice, Poland). Hydrochloric acid, phosphoric acid, and sodium hydroxide were purchased from Merck (Darmstadt, Germany). Deionized water was employed in the preparation of the solutions. The nitrogen gas was purchased from Linde (technical nitrogen 4.0, Linde Gaz Poland, Krakow, Poland).

### 3.2. Carbonaceous Adsorbents Characteriztaion

The residues from the extraction of the fungus *Inonotus obliquus*, which had a moisture level of 8.5 wt.% and a grain size of 0.80–1.75 mm, underwent pyrolysis in a microwave oven (Phoenix, CEM Corporation, Matthews, IL, USA). The heating process was performed under a nitrogen atmosphere (170 mL/min) at two different temperatures: 500 °C (M5) and 600 °C (M6). The samples were thermostated for 60 min in each case. Initially, the starting material was placed in the microwave oven. The furnace was programmed to heat at a rate of 10 °C/min. Once the desired temperature was reached, the precursor samples were thermostated and cooled with nitrogen flow until they reached room temperature.

As the ash components block some of the pores in the structure of the carbonaceous material, resulting in low specific surface areas of the carbonaceous sorbents, a washing process was carried out for the carbonaceous adsorbents. The carbon adsorbents were rinsed with hydrochloric acid and hot distilled water until the filtrate pH level was neutral. Subsequently, the samples were dried to achieve a constant weight at 110 °C.

### 3.3. Preparation of Activated Carbons

The elemental composition of the precursor and carbonaceous adsorbents were measured by a Thermo Scientific FLASH 2000 Elemental Analyzer, Langenselbold, Germany. The acid–base properties of the obtained samples were characterized by measurement of pH of their water extracts and determination of the acidic and basic functional groups by the Boehm titration method [7]. The specific surface areas of the adsorbents and their porous structure were determined by the low-temperature nitrogen adsorption/desorption method. The isotherm of nitrogen adsorption was recorded at 77 K by an analyzer AutosorbiQ instrument, provided by Quantachrome Instruments (Boynton Beach, FL, USA). Prior to measurements, the samples were degassed for 12 h at 300 °C. S_BET_ values were estimated using the standard Brunauer–Emmett–Teller equation for nitrogen adsorption data in the range of relative pressure p/p_0_ from 0.05 to 0.30. The pore size distribution and total pore volume were determined on the basis of the BJH (Barrett–Joyner–Halenda) model.

### 3.4. Adsorption Study

The sorption capacities of the samples for NO_2_ were assessed using an electrochemical gas concentration monitor, the QREA PLUS model PGM-2000. A glass reactor containing the carbonaceous adsorbent (3 mL) and a blend of NO_2_ and air was directed through the adsorbent bed. To achieve a NO_2_ concentration of 1000 ppm, the air and NO_2_ were mixed in specific ratios (NO_2_ 90 mL/min, air 360 mL/min). This adsorption process took place under both dry and wet (70% humidity) conditions. Additionally, to investigate the impact of moisture on the samples’ sorption capacities, they were subjected to prehumidization with humid air (70% humidity) for 30 min. Subsequently, adsorption tests were conducted again under dry/wet conditions. After completion of the adsorption phase, the desorption rate of NO_2_ from the adsorbent bed was quantified [12].

The study conducted adsorption tests on carbonaceous adsorbents with aqueous solutions of methylene blue and malachite green. The procedure involved preparing standard solutions of organic dyes with a concentration of 1000 mg/L in the initial stage. From these solutions, working solutions were prepared, and then flat-bottom flasks were filled with 50 mL of a methylene blue/malachite green solution with various concentrations. A measure of 20 mg aliquots of a given adsorbent were placed in the flasks. Materials with a particle size of 0.08 mm were employed for labeling. The specimens were shaken for 8 h on a rotary shaker (Heidolph, Schwabach, Germany) at a speed of 300 rpm. Subsequently, the samples were separated from the solvent using a Frontiner™ FC5515 laboratory centrifuge (OHAUS, Parsippany, NJ, USA). Absorption readings were taken for each filtered solution using a double-beam UV–Vis Carry 100 Bio spectrometer (Agilent, Santa Clara, CA, USA). The absorbance of methylene blue was recorded at a wavelength of 665 nm and for malachite green at 663 nm. Final concentrations of the dye were calculated based on a standard curve, and the sorption capacity of the samples was determined.

The sorption capacities of samples M5 and M6 were calculated based on Equation (1):

(1) $$q_{e}=\frac{C_{0}-C_{e}}{m}\times V$$

where C_0_ is the initial concentration of methylene blue/malachite green (mg/g), C_e_ represents the concentration of methylene blue/malachite green in the solution at equilibrium (mg/g), m is the total mass of the adsorbent M5/M6 (g), and V is the volume of methylene blue/malachite green solution (L).

In this study, several adsorption isotherm models were employed to characterize the relationship between the surface of M5/M6 test samples and the dyes methylene blue and malachite green. These models consisted of the Langmuir, Freundlich, Temkin, and Dubinin–Raduszkevich models.

Langmuir [21] hypothesized that there exists a defined number of active sites on the surface of the adsorbent, and each site can only bind one molecule of the adsorbate. The adsorbed substance’s molecules create a monomolecular layer and exhibit no mutual interactions. The Langmuir equation is formulated as follows (2):

(2) $$\frac{C_{e}}{q_{e}}=\frac{1}{K_{L}\times q_{max}}+\frac{C_{e}}{q_{max}}$$

where K_L_ is the Langmuir adsorption constant (L/mg), and q_max_ is the maximum adsorption capacity of the monolayer (mg/g).

On the other hand, Freundlich’s theory [22] proposes that multilayer adsorption (3) can occur on the surfaces of adsorbents:

(3) $$lnq_{e}=lnK_{F}+\frac{1}{n}lnC_{e}$$

where K_F_ is the Freundlich constant (mg/g(L/mg)^1/n^), and 1/n is the constant related to the adsorbate’s affinity to the adsorbent, known as the heterogeneity factor.

To explain the linear relationship between surface coverage and the heat of adsorption (4), the Temkin isotherm model [24] is employed:

(4) $$lnq_{e}=BlnA_{T}+BlnC_{e}$$

where B is a constant related to the heat of adsorption (J/mol) and A_T_ is an empirical constant related to the maximum binding energy (L/mg).

The Dubinin–Raduszkevich model [27] is one of the models employed in this study.

This model is based on the pore-filling mechanism and assumes a heterogeneous surface with variable adsorption potential (5):

(5) $$lnq_{e}=lnq_{max}-\beta \varepsilon^{2}$$

where ε is the Polanyi potential, and β is a constant related to the adsorption energy, which allows for the calculation of free energy in units of mol^2^/kJ^2^. E represents the free energy in kJ/mol (6):

(6) $$E=\frac{1}{\sqrt{2\beta }}$$

Contact time optimization was conducted on a 50 mL solution of methylene blue and malachite green, with a concentration of 40 mg/L and an adsorbent mass of 20 mg. The sample shaking speed was maintained at 300 rpm, and the process was carried out for a period of 6 h. The study enabled the calculation of kinetic parameters [17], specifically for the pseudo-first order (7) and pseudo-second order (8) models:

(7) $$\log\left(q_{e}-q_{t}\right)=logq_{e}-\frac{k_{1}t}{2.303}$$

(8) $$\frac{t}{q_{t}}=\frac{1}{k_{2}{q_{e}}^{2}}+\frac{t}{q_{e}}$$

where k_1_ is the kinetic constant of the pseudo-first-order model (1/min) and k_2_ is the second-order rate constant (g/mol·min).

The influence of the pH of the methylene blue/malachite green solution on the adsorption capacity of the carbon adsorbent was assessed across a pH range of 2 to 12. The measurements were conducted over a period of 6 h, using 20 mg of the sample and 50 mL of a dye solution with a concentration of 40 mg/L.

Additionally, sorption experiments for methylene blue/malachite green on the prepared carbonaceous adsorbents were conducted at temperatures of 298, 318, and 338 K (9–11):

(9) $$∆G{}^{\circ}=-RTlnK_{d}$$

(10) ∆G°=∆H°−T∆S°

(11) $$lnK_{d}=\frac{∆S{}^{\circ}}{R}-\frac{∆H{}^{\circ}}{RT}$$

where: ΔG°–Gibbs free energy (kJ/mol), R—gas constant (J/mol·K), T—temperature (K) and K_d_—thermodynamic equilibrium constant, ΔH°—enthalpy change (J/mol) and ΔS°—entropy change (J/mol·K).

## 4. Conclusions

Studies carried out in this work illustrate the practical potential for obtaining efficient carbon adsorbents for removing harmful gaseous and liquid pollutants through the use of microwave pyrolysis at a relatively low temperature, without the need for additional activation methods. Furthermore, the adsorbents received were obtained from the remaining extract of the *Inonotus obliquus* mushroom, thus making it possible to value this waste and reduce the costs of producing carbonaceous adsorbents. The research conducted showed that:

(1) Microwave heating enabled the pyrolysis of the raw material, producing carbonaceous materials with a surface area of 372 and 502 m^2^/g and a micro-mesoporous structure.
(2) The synthesized carbonaceous adsorbents featured both acidic and basic groups on their surface. However, the basic groups dominated the surface character.
(3) The efficacy of the developed carbonaceous adsorbents in removing nitrogen oxide(IV) has been demonstrated, despite their low specific surface area. This was especially observed when the adsorption process occurred under wet conditions and was preceded by a 30-min flushing of the adsorbent bed with moist air.
(4) The efficiency of the carbonaceous adsorbents in removing organic dyes increased with the initial concentration of the dye solution. The adsorption rate followed pseudo-second order kinetics. Langmuir-type isotherms were observed in all experiments on the adsorption of methylene blue/malachite green.

## Figures and Tables

**Table 1:** Elemental analysis (wt.%) and acid–base properties of the precursor and carbonaceous adsorbents.

Sample	C^daf^	H^daf^	N^daf^	S^daf^	O^daf^ *	Ash	Acidic Groups (mmol/g)	Basic Groups (mmol/g)	pH
Precursor	55.5	8.2	2.9	0.1	33.3	6.2	1.5	1.1	6.8
M5	73.7	2.7	3.8	0.2	19.6	9.1	0.3	2.8	10.5
M6	79.9	2.5	3.1	0.2	14.3	9.8	0.2	3.1	10.9

^daf^—dry-ash-free basis; *—determined by difference.

**Table 2:** Textural parameters of the carbonaceous adsorbents obtained.

Carbonaceous Adsorbent	Surface Area (m^2^/g)	Micropore Area (m^2^/g)	Total Pore Volume (cm^3^/g)	Micropore Volume (cm^3^/g)	Average Pore Diameter (nm)
M5	372	268	0.5	0.4	3.7
M6	502	415	0.6	0.5	3.2

**Table 3:** NO_2_ breakthrough capacities of carbonaceous adsorbents obtained (mg/g).

Sample	Dry Conditions	Mix Dry Conditions	Wet Conditions	Mix Wet Conditions
M5	8.9	10.3	19.0	25.7
M6	10.0	12.0	23.9	28.2

**Table 4:** Isotherm study of methylene blue and malachite green.

Isotherms	Parameters	Methylene Blue		Malachite Green	
		M5	M6	M5	M6
Langmuir	q_e_ (mg/g)	100	107	73	76
	R^2^	0.999	0.999	0.999	0.997
	q_max_	101	109	75	77
	K_L_ (L/mg)	0.05	0.17	0.06	0.07
Freundlich	R^2^	0.961	0.978	0.811	0.702
	K_F_ (mg/g(L/mg)^1/n^)	68.6	92.7	48.1	67.3
	1/n	0.22	0.08	0.27	0.04
Temkin	R^2^	0.369	0.472	0.908	0.968
	B (J/mol)	138.6	91.8	214.5	225.2
	A_T_ (L/mg)	19.5	9.2	67.2	68.9
Dubinin–Raduskevich	R^2^	0.922	0.934	0.993	0.986
	q_max_ (mg/g)	90	103	68	72
	E (kJ/mol)	3.73	6.06	3.70	3.33

**Table 5:** Kinetic parameters for adsorption of methylene blue/malachite green.

Model	Parameters	Methylene Blue		Malachite Green	
		M5	M6	M5	M6
pseudo-first order	q_t_ (mg/g)	83	85	66	70
	q_e,cal_ (mg/g)	30	16	47	26
	R^2^	0.517	0.665	0.914	0.745
	k_1_ (1/min)	2.99 × 10^−3^	8.98 × 10^−3^	1.59 × 10^−2^	1.31 × 10^−2^
pseudo-second order	q_e,cal_ (mg/g)	87	88	67	71
	R^2^	0.995	0.999	0.999	0.999
	k_2_ (g/mg × min)	4.64 × 10^−4^	8.43 × 10^−4^	6.94 × 10^−4^	1.26 × 10^−3^

**Table 6:** Thermodynamic parameters of the adsorption of methylene blue/malachite green on the carbonaceous adsorbents.

Biocarbon	Dyes	q_e_ (mg/g)	Temperature (K)	∆G° (kJ/mol)	∆H° (kJ/mol)	∆S° (J/mol K)
M5	methylene blue	70	298	−6.2	7.4	45.6
		77	318	−7.2		
		80	338	−8.00		
M6		73	298	−7.9	6.8	49.2
		79	318	−8.7		
		82	338	9.9		
M5	malachite green	19	298	−3.3	25.4	96.0
		35	318	−5.2		
		60	338	−7.2		
M6		23	298	−3.8	21.4	85.2
		47	318	−6.0		
		61	338	−7.1		

**Table 7:** NO_2_/methylene blue/malachite green sorption capacity of selected adsorbents.

Adsorbent/Precursor	Adsorbent	Adsorption Capacity (mg/g)	References
nettle seeds	NO_2_	59.1	[5]
olive stones		131	[19]
waste tires		11.44	[20]
M6		28.2	This study
orange and lemon skins	Methylene blue	33	[21]
Maghara coal		28.09	[22]
Sargassum (sp.)		223	[23]
M6		107	This study
plantain leaves	Malachite green	266.80	[24]
date stones		98	[25]
Limonia acidissima (wood apple)		34.56	[26]
M6		76	This study

## Data Availability

Data is contained within the article.
